# Responses of cut-lily quality and rhizosphere soil microecology to different microbial inoculants

**DOI:** 10.3389/fmicb.2026.1962117

**Published:** 2026-10-06

**Authors:** Yingdong Yang, Lihong Zhou, Ruiqi Zhang, Xinying Hu, Weidong Wang, Xueyan Li, Yiguang Bai, Muhammad Irfan, Yijun Chen, Lijing Chen

**Affiliations:** 1 Institute of Flowers, Liaoning Academy of Agricultural Sciences, Key Laboratory of Flower Science of Liaoning Province, Shenyang, Liaoning, China; 2 College of Bioscience and Biotechnology, Key Laboratory of Agriculture Biotechnology, Key Laboratory of Protected Horticulture (Ministry of Education), Shenyang Agricultural University, Liaoning, China; 3 Department of Biotechnology, University of Sargodha, Sargodha, Pakistan

**Keywords:** co-occurrence network, lily, microbial inoculants, rhizosphere microbial community, soil physicochemical properties, *Trichoderma*

## Abstract

Continuous cropping obstacles and soil-borne diseases have long constrained the development of the cut lily industry, and microbial inoculants have become an important tool for achieving green production. To screen for efficient inoculants suitable for cut lily and to reveal the mechanisms of action, this study used the dominant cultivar ‘Siberia’ (*Lilium* ‘Siberia’) as material and conducted a field experiment under solar greenhouse conditions in Lingyuan, Liaoning Province, using three commercial microbial inoculants: Treatment A (Shentai *Trichoderma* combination, containing *Trichoderma harzianum* and its metabolites), Treatment B (Moon composite inoculant, containing *Trichoderma*, *Purpureocillium lilacinum*, *Bacillus subtilis*, etc.), and Treatment C (Fengde composite inoculant, mainly *Bacillus subtilis*). The study systematically measured lily growth and commercial traits, soil physicochemical properties, and rhizosphere microbial community structure, and analyzed the linkage mechanism of “inoculant—soil physicochemical properties—rhizosphere microbial community—comprehensive lily quality.” The results showed that Treatment A (Shentai *Trichoderma* combination) had the best comprehensive effect, significantly increasing plant height, stem diameter, and green leaf rate, and reducing leaf yellowing and abscission index. At the same time, it significantly increased soil total nitrogen, total phosphorus, available nitrogen, and organic matter content, and optimized the rhizosphere microecological environment. Further microbial community analysis indicated that Treatment A specifically enriched putatively beneficial taxa such as *Pseudomonas* and *Streptomyces*, and functional fungi such as *Trichoderma* and *Mucor*, while reducing the relative abundance of potentially pathogenic taxa such as *Fusarium* and *Rhizoctonia*. It also increased the modularity and proportion of positive correlations in the bacterial-fungal co-occurrence network, forming a stable growth-promoting and disease-resistant functional module. This study clarifies the core effects of the *Trichoderma*-based inoculant on lily growth, soil improvement, and rhizosphere microecological regulation, suggests a mechanism by which it synergistically improves cut lily quality and stress resistance through optimizing soil nutrients and reshaping microbial community structure, identifies a promising inoculant formulation with potential for green lily production, and provides a theoretical basis and technical support for the sustainable development of the cut lily industry.

## 1 Introduction

Lilies (*Lilium* spp.) are important ornamental flowers worldwide, their cut flowers are highly prized by consumers for their elegant form, rich variety of colors, and auspicious symbolism, and held significant market value (Ferrante et al., 2011). China is the world’s largest producer of cut lilies, and ‘Siberia’ is a representative variety of the Oriental lily hybrid series, Plants can grow to a height of 100–120 cm, features white petals and a delicate fragrance, and holds a significant position in the market (Zheng et al., 2014). As China’s economy continues to grow and its residents’ income levels rise, the domestic flower consumption market is expanding rapidly (Luo and Liao, 2020). However, as the lily cut flower industry expands on a large scale, issues such as the deterioration of soil physicochemical properties and the frequent occurrence of soil-borne diseases-resulting from years of continuous cropping-have become increasingly prominent, severely limiting improvements in the quality and yield of lily cut flowers (Qin et al., 2025a; Zhang et al., 2025). Excessive use of chemical fertilizers and pesticides not only increases production costs but also leads to a range of problems, including an imbalance in soil microbial communities, environmental pollution, and pesticide residues in agricultural products (Zhou et al., 2019). Microbial communities, acting as the “driving force” of soil nutrient cycling, can regulate soil physicochemical properties by secreting metabolites, activating inert nutrients, and inhibiting the colonization of pathogens, thereby influencing plant growth and stress tolerance (Philippot et al., 2024). In recent years, with the widespread adoption of the concept of sustainable green agriculture. Microbial inoculants show great promise as an environmentally friendly biological control method for improving crop growth, enhancing stress tolerance, and restoring soil health. (Díaz-Rodríguez et al., 2025; Chen et al., 2021).

Beneficial rhizosphere microorganisms can strengthen the root system, improve nutrient uptake, increase tolerance to abiotic stress, and activate the plant immune system (Bakker et al., 2020; Kumar and Dubey, 2020). As an environmentally friendly, efficient, and safe biofertilizer, microbial inoculants can improve soil fertility, promote plant growth, enhance stress tolerance, and subsequently increase crop yield and quality through multiple mechanisms, including nitrogen fixation, phosphate solubilization, potassium solubilization, secretion of phytohormones, induction of systemic resistance, and antagonism of phytopathogens (Tan et al., 2025). Previous studies have shown that *Trichoderma* and *Bacillus* can significantly suppress soil-borne pathogens while promoting the activation of soil nitrogen, phosphorus, and potassium (Xiao et al., 2025). In recent years, beneficial microorganisms such as *Trichoderma*, *Bacillus*, and *Pseudomonas* have demonstrated favorable growth-promoting and biocontrol effects in vegetables and cereal crops (Liu et al., 2026a; Ma et al., 2023; Fröhlich et al., 2012). In horticultural systems, these microorganisms enhance flower production and quality by producing plant hormones, solubilizing nutrients, fixing atmospheric nitrogen, and protecting plants from biotic and abiotic stresses (Sahu et al., 2025; Tang et al., 2026). However, research on the application of microbial inoculants in cut lily production remains relatively limited. Existing studies tend to focus on single inoculants or individual growth indicators (Liu et al., 2026b; Qin et al., 2025b), lacking comprehensive analyses of the interplay among “inoculant–soil physicochemical properties–rhizosphere microbial community–plant quality.” Moreover, the effects and underlying mechanisms of different inoculant formulations on lily growth and soil microecosystems may vary considerably due to differences in microbial composition, concentration, and application methods. The impacts of inoculant application on soil physicochemical properties, microbial community structure, and rhizosphere microecology therefore warrant further investigation.

In this study, the major cut lily cultivar ‘Siberia’ (*Lilium* ‘Siberia’) was used as experimental material. A field plot experiment was conducted in a solar greenhouse in Lingyuan City, Liaoning Province—a major lily-producing region—using three commercial microbial inoculants: Treatment A, Shentai *Trichoderma* combination; Treatment B, Moon composite inoculant; and Treatment C, Fengde composite inoculant. By assessing multiple parameters related to lily growth performance, soil physicochemical properties, and rhizosphere microbial community structure, this study aimed to compare the regulatory effects of different inoculants on rhizosphere microbial community structure, identify key functional microbial taxa and soil physicochemical factors influencing lily growth, elucidate the intrinsic linkage mechanism underlying the “inoculant–soil physicochemical properties–rhizosphere microbial community–comprehensive plant quality” continuum, and screen for optimal inoculant formulations suitable for the green production of cut lilies. These findings provide a theoretical basis and practical guidance for the sustainable development of the cut lily industry.

## 2 Materials and methods

### 2.1 Experimental materials and soil

The selected plant material was lily (*Lilium* ‘Siberia’), with Dutch imported bulbs of 16–18 cm in circumference, totaling 7,560 bulbs. Bulbs were germinated before planting, at a germination temperature of 10 ± 1 °C, and planted when the bud length reached 5–7 cm. The experiment was conducted in a solar greenhouse of Kaisheng Agricultural Professional Cooperative, a cooperative enterprise of Liaoning Academy of Agricultural Sciences located in the National Modern Agricultural Industrial Park, Xiaochengzi Town, Lingyuan City, Chaoyang City, Liaoning Province. The soil type was loam soil representative of the local area. Before planting, 0–20 cm soil samples were randomly collected from multiple points in the test area and marked as BFP. Basic physicochemical indexes including soil pH, electrical conductivity (EC), organic matter (OM), available nitrogen (AN), available phosphorus (AP) and available potassium (AK) were measured to provide basic data for subsequent analysis of the regulatory effect of inoculants on soil fertility (Table 1).

**Table 1:** Effects of different inoculant treatments on rhizosphere soil physicochemical properties of lilies.

Index	pH	TN (g/kg)	AN (mg/kg)	TP (g/kg)	AP (mg/kg)	TK (g/kg)	AK (mg/kg)	OM (g/kg)
BFP	7.69 ± 0.07^a^	1.16 ± 0.03^b^	62.76 ± 0.98^b^	0.79 ± 0.01^b^	68.51 ± 1.48^b^	12.26 ± 0.65^a^	308.20 ± 4.15^c^	18.33 ± 2.04^b^
CK	6.70 ± 0.29^ab^	1.89 ± 0.19^ab^	111.65 ± 11.69^ab^	0.81 ± 0.02^b^	121.66 ± 36.25^a^	13.33 ± 0.43^a^	380.33 ± 88.31^bc^	42.70 ± 4.19^ab^
A	7.00 ± 0.18^ab^	2.56 ± 0.11^a^	154.73 ± 4.03^a^	1.00 ± 0.03^a^	107.73 ± 3.96^ab^	12.55 ± 0.35^a^	385.53 ± 3.81^bc^	48.95 ± 3.37^a^
B	6.51 ± 0.65^b^	2.51 ± 1.11^a^	149.90 ± 66.53^a^	0.82 ± 0.03^b^	100.13 ± 32.43^ab^	12.62 ± 0.37^a^	436.70 ± 6.76^ab^	59.53 ± 4.09^a^
C	6.70 ± 0.53^ab^	2.46 ± 0.38^a^	154.58 ± 26.46^a^	0.83 ± 0.03^b^	100.45 ± 9.17^ab^	12.98 ± 0.52^a^	462.80 ± 7.18^a^	48.69 ± 8.45^ab^

Values are mean ± standard deviation (*n* = 3 replicate plots). Different lowercase letters in the same column indicate significant differences among treatments (one-way ANOVA, Duncan’s test, *p* < 0.05). BFP: Soil before planting; CK: Control without inoculant; A, B, C: See Table 2. TN: Total nitrogen; AN: Available nitrogen; TP: Total phosphorus; AP: Available phosphorus; TK: Total potassium; AK: Available potassium; OM: Organic matter.

### 2.2 Experimental microbial agents

#### 2.2.1 Experimental design

The field trial ran from October 30, 2024 (when the bulbs were planted) to March 15, 2025 (when the cut flowers were harvested). The trial used a randomized block design with a single factor: different microbial agent treatments. Four treatment groups were established: A (Treatment A: Liaoning Shentai Trichoderma mixture), B (Treatment B: Guangzhou Muen composite fungicide), C (Treatment C: Dalian Fengde composite fungicide), and CK (control: conventional field management). Three commercial microbial inoculants were used in this study. Detailed information on product names, manufacturers, active ingredients, viable-count concentrations, and effective microorganism count/active ingredient content formulations is provided in Table 2.

**Table 2:** Species, manufacturers, active ingredients, and content of the three different microbial inoculants.

Inoculant label	Product name	Manufacturer	Active ingredients	Effective microorganism count/active ingredient content
Treatment A (A)	① Cici Sheng ② Inducer	Liaoning Shentai Microbial Technology Co., Ltd	① *Trichoderma harzianum* ② *Trichoderma* metabolites	① 1.0 × 10^9^ CFU/g ② 100%(pure metabolites)
Treatment B (B)	① Mujianda ② Junhudun ③ Junyangyuan	Moon (Guangzhou) Biotechnology Co., Ltd	① *Trichoderma harzianum* *Purpureocillium lilacinum* *Bacillus subtilis* ② *Penicillium bilaiae* ③ *Trichoderma harzianum*	① 1.0 × 10^9^ CFU/g ② 2.0 × 10^8^ CFU/g ③ 5.0 × 10^8^ CFU/g
Treatment C (C)	Yingfeng	Fengde Biotechnology (Dalian) Co., Ltd.	Composite bacteria (mainly *Bacillus subtilis*)	Composite bacteria 65% (mainly *Bacillus subtilis*, accounting for 16% of the total mass); amino acids 30%; trace elements 5%

CFU, colony-forming units. Treatments A, B and C correspond to three commercial microbial inoculants. CK (control) was not applied with inoculants but received the same field management.

Each treatment had three replicates, each replicate being one experimental plot. Each plot contained three cultivation beds, with an area of 30 m^2^. The total area per treatment was 90 m^2^ (3 plots × 30 m^2^/plot), and the total experimental area was 360 m^2^ (4 treatments × 90 m^2^/treatment). Bulbs were planted in three rows per bed with a spacing of 10 cm × 25 cm; each bed was planted with 210 bulbs to ensure consistent planting density across plots.

Inoculant application dosage, timing, and method for each treatment followed the product instructions and are documented in detail in Supplementary Table S1. The control group (CK) received conventional field management without inoculants, including irrigation and fertilization; full details of fertilizer types, amounts, and application dates for all treatments are provided in Supplementary Table S1. All other environmental conditions (temperature, humidity, light, irrigation amount) were kept consistent among treatments except for the inoculant difference.

### 2.3 Experimental items and methods

#### 2.3.1 Measurement of lily growth indicators

Thirty representative lilies were randomly selected from each plot, numbered 1–30 and tagged. The same samples were continuously tracked and measured at the budding stage, before cut flower harvesting and after harvesting. During the growth period, the number of flower buds, plant height, stem diameter, maximum flower bud length, top leaf length, top leaf width, chlorophyll content (SPAD value), total number of leaves, number of green leaves, number of fallen leaves and number of yellow leaves were measured, and green leaf rate, defoliation rate and yellow leaf rate were calculated. After cut flower harvesting, bulb circumference and bulb fresh weight were measured. All morphological indexes were measured according to standard methods: plant height was accurate to 0.1 cm, stem diameter and flower bud length to 0.01 mm, bulb circumference to 0.1 cm, and fresh weight to 0.01 g. Chlorophyll content was measured on functional leaves using a SPAD-502 chlorophyll meter. Healthy green leaves were defined as grade 0, mild yellow leaves as grade 1, and severe defoliation as grade 2. The formula of leaf yellowing and abscission index (LYAI):

$$\begin{array}{l} \text{LYAI}=(\begin{array}{l} \text{Number of yellow leaves}\times 1\\ +\text{Number of fallen leaves}\times 2 \end{array})\\ /\text{Total number of leaves}\times 100. \end{array}$$

#### 2.3.2 Soil sample collection and analysis

After cut flower harvesting, rhizosphere soil samples were collected from each treatment. Briefly, the entire root system of tagged plants was excavated, and loosely adhering soil was removed by gentle shaking. Soil firmly attached to the root surface (approximately 1–2 mm) was collected as rhizosphere soil using a sterile brush. For each treatment, soil from multiple plants within a replicate plot was pooled to obtain a composite sample of approximately 1 kg; four composite samples (one per treatment) were collected in total. A portion of each fresh soil sample was immediately frozen at −80 °C for microbial community analysis, and the remainder was air-dried, ground, and sieved for physicochemical analysis. Total nitrogen (TN), total phosphorus (TP), available nitrogen (AN), available phosphorus (AP), pH, calcium (Ca), magnesium (Mg), copper (Cu), iron (Fe), zinc (Zn), total potassium (TK), available potassium (AK), and organic matter (OM) were measured. The methods used for each parameter were as follows: Total nitrogen was determined using the sulfuric acid digestion-Kjeldahl method; Quick-acting nitrogen was determined using the alkaline diffusion method; Total phosphorus was determined using the H_2_SO_4_-HClO_4_ acid digestion–molybdenum-antimony complex spectrophotometric method; Quick-acting phosphorus in neutral and calcareous soils was determined using the sodium bicarbonate extraction-molybdenum-antimony colorimetric method; pH was determined using the potentiometric method; Ca, Mg, Cu, Fe, and Zn were determined using the nitric acid-hydrochloric acid-hydrofluoric acid microwave digestion-ICP-MS method; Total potassium was determined using the NaOH alkali fusion–flame photometry method; Quick-acting potassium was determined using ammonium acetate extraction followed by flame photometry; Organic matter was determined using potassium dichromate oxidation followed by external heating.

### 2.4 DNA extraction and illumina sequencing

After cut lily harvesting, soil near the stem roots of tagged samples was collected, immediately frozen in a −80 °C refrigerator, and transported to Guangzhou Genedenovo Biotechnology Co., Ltd. with dry ice for 16S and ITS detection. Genomic DNA was extracted using HiPure Soil DNA Kits. The concentration and purity of extracted DNA were detected by NanoDrop 2000 (A260/A280 ratio 1.8–2.0, A260/A230 ratio about 2.2), and the integrity was verified by agarose gel electrophoresis. The V3–V4 region of bacterial 16S rRNA gene was amplified by PCR using primers 341F (5′-CCTACGGGNGGCWGCAG-3′) and 806R (5′-GGACTACHVGGGTATCTAAT-3′). The ITS1 region of fungi was amplified by PCR using primers ITS1_F_KYO2 (5′-TAGAGGAAGTAAAAGTCGTAA-3′) and ITS86R (5′-TTCAAAGATTCGATGATTCAC-3′). The PCR reaction system was 50 μL, using Q5 high-fidelity enzyme system. The reaction procedure was: pre-denaturation at 95 °C for 5 min; denaturation at 95 °C for 1 min, annealing at 60 °C for 1 min, extension at 72 °C for 1 min, 30 cycles; final extension at 72 °C for 7 min. PCR products were purified by AMPure XP Beads and quantified using Qubit 3.0. The sequencing library was constructed using Illumina DNA Prep Kit. After the library quality was qualified by ABI StepOnePlus Real-Time PCR system, sequencing was performed on Illumina Novaseq 600 platform in PE250 mode. All the above operations were completed in Guangzhou Genedenovo Biotechnology Co., Ltd.

### 2.5 Data analysis

All data were initially statistically calculated using Microsoft Excel software. One-way analysis of variance (ANOVA) was performed on the data using SPSS software, and multiple comparisons of significant differences were conducted using Duncan’s test (significance level *p* < 0.05). The results were expressed as mean ± standard deviation (m ± SD). Microbial data analysis including alpha diversity, beta diversity, community composition, linear discriminant analysis effect size (LEfSe) analysis, network construction and Mantel test was performed using QIIME2 and R language. Graphs were drawn using Origin 2025 and Gephi software to intuitively show the differences of each treatment in plant growth, cut flower quality and soil microbial community, and finally a comprehensive evaluation of the effect of inoculants on cut lily quality improvement was obtained.

## 3 Results

### 3.1 Effects of different microbial inoculant on cut lily quality

To evaluate the effects of the three inoculant formulations on cut lily quality, growth-related traits and the leaf yellowing and abscission index (LYAI) were measured. Among them, plant height, stem diameter and chlorophyll content differed significantly among treatments (*p* < 0.05), whereas the remaining traits did not (*p* > 0.05). Treatment A exhibited the strongest and most consistent growth-promoting effect among the three inoculants (Figures 1A,B). It produced the greatest plant height (111.70 ± 2.30 cm), significantly exceeding CK (100.64 ± 1.78 cm; *p* < 0.05), and the greatest stem diameter (7.32 ± 0.08 mm), also significantly exceeding CK (6.62 ± 0.09 mm; *p* < 0.05). Notably, Treatment A was the only inoculant that significantly outperformed CK in both plant height and stem diameter, whereas Treatment B was superior only in plant height (107.46 ± 1.89 cm) and Treatment C only in stem diameter (7.06 ± 0.11 mm). Treatment A also showed the smallest interquartile range for plant height, further indicating the most stable growth-promoting effect. For chlorophyll content (Figure 1D), Treatment B showed the highest SPAD value (71.78 ± 1.49), significantly higher than CK (65.04 ± 1.03) and Treatment A (62.92 ± 1.89; *p* < 0.05), while Treatment C (67.36 ± 2.76) was intermediate. This indicates that the three inoculants differ in their functional emphasis, with Treatment A favoring growth and stem quality and Treatment B favoring chlorophyll accumulation. For flower bud number, flower bud length, top leaf length, top leaf width, bulb circumference, bulb fresh weight, green leaf rate, yellow leaf rate and defoliation rate, no significant differences were detected among treatments (*p* > 0.05; Figures 1C,E–G; Supplementary Figure S1). Nevertheless, Treatment A consistently maintained numerically higher green leaf rate and numerically lower yellow leaf rate and defoliation rate than CK, suggesting a favorable trend in leaf retention and senescence delay. The leaf yellowing and abscission index (LYAI) was lowest under Treatment A (53.67), followed by B (54.67), CK (57.00) and C (61.00), but no significant differences were detected among treatments (*p* > 0.05; Figure 1H). The lowest LYAI under Treatment A is consistent with its numerically higher green leaf rate and lower yellow leaf rate and defoliation rate, further supporting its advantage in maintaining leaf vitality.

**Figure 1:**
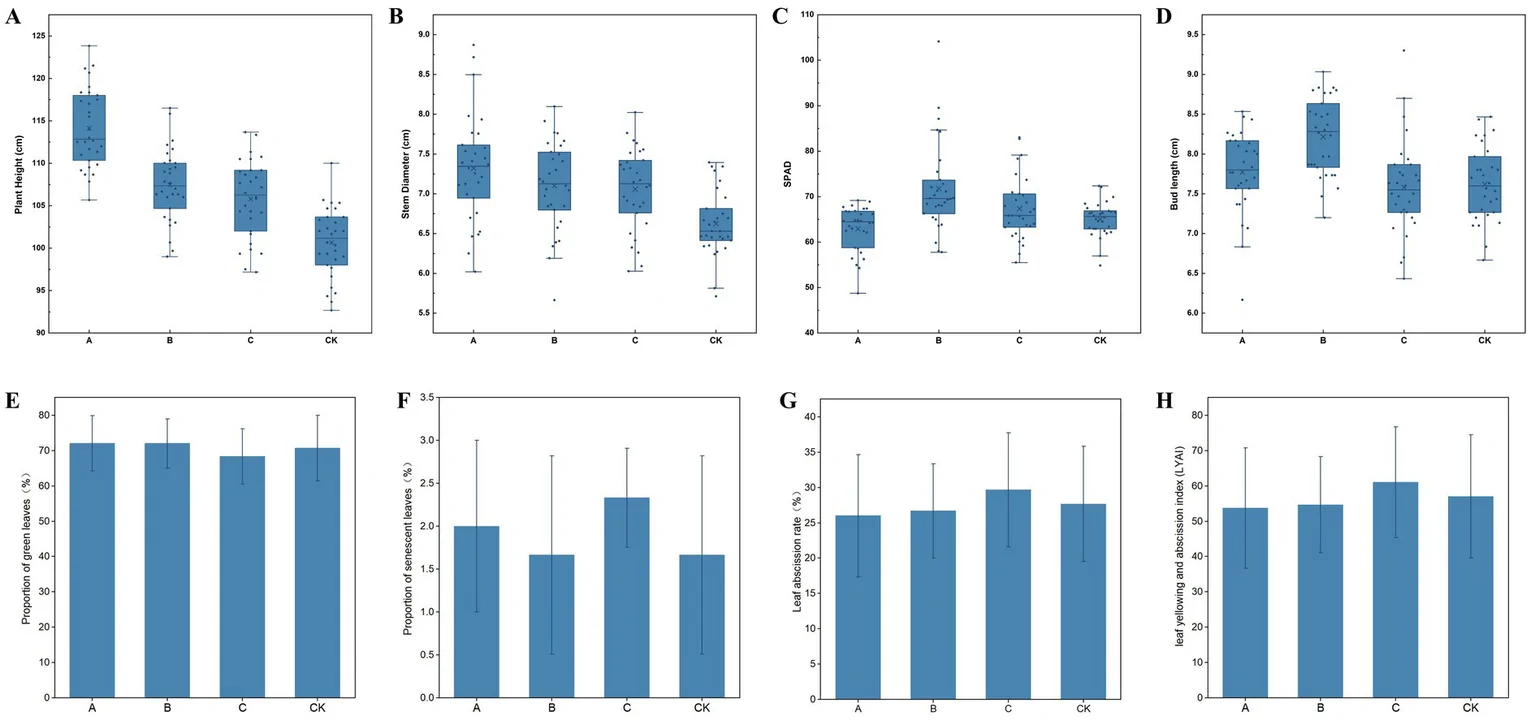
Effects of different inoculant treatments on lily growth indexes and leaf yellowing and abscission index. **(A)** Plant height; **(B)** Stem diameter; **(C)** Flower bud length; **(D)** Leaf SPAD value; **(E)** Green leaf rate; **(F)** Yellow leaf rate; **(G)** Defoliation rate; **(H)** leaf yellowing and abscission index. **(A–D)** are box plots showing median, 25th–75th percentiles (box), and minimum–maximum values (whiskers). **(E–H)** are bar charts showing mean ± standard deviation (*n* = 30 plants per treatment).

### 3.2 Effects of different microbial inoculants on soil physicochemical properties

The intergroup differences in lily growth and leaf yellowing and abscission index suggested that different microbial inoculant treatments might exert different effects by regulating soil environment and microbial community. To further analyze the internal mechanism of the comprehensive advantages of Treatment A, soil physicochemical properties under different inoculant treatments were determined. Treatment A had the most prominent comprehensive optimization effect on soil physicochemical properties (Table 1). Before planting, the basic soil (BFP group) was alkaline, with medium to low contents of TN, AN, TP, AP and OM, indicating low soil nutrient content that required exogenous supplementation to meet lily growth needs. After planting, the soil pH of the control group without inoculant (CK group) decreased significantly from 7.69 before planting to 6.70, tending to be neutral, which was more suitable for lily growth (Yang, 2024). Meanwhile, its available nutrients were significantly improved: AN, AP, AK and OM increased by 77.8, 77.5, 23.4 and 132.8%, respectively, compared with before planting, indicating that root activities during lily growth could effectively promote soil nutrient activation, while total nutrients such as TN, TP and TK fluctuated slightly, suggesting limited consumption of soil basic total nutrients by lilies. Under inoculant treatments, Treatment A had the best effect: its contents of TN, TP and AN increased by 35.45, 23.46 and 38.67%, respectively, compared with CK group, with particularly prominent soil phosphorus activation capacity, and OM content further increased to 48.95 g/kg, significantly higher than CK group. Treatments B and C had different focuses in soil regulation: Treatment B significantly improved soil AK (436.70 mg/kg) and OM (59.53 g/kg) contents, and Treatment C had the strongest enrichment effect on soil AK (462.80 mg/kg) but weak phosphorus activation capacity. In conclusion, lily planting itself can significantly optimize soil available nutrient status, and Treatment A can further synergistically improve key soil nutrient contents such as nitrogen and phosphorus, providing a more balanced and suitable soil environment for lilies.

### 3.3 Regulation of the rhizosphere microbial community by soil physicochemical parameters

The results above showed that microbial inoculant A significantly improved rhizosphere soil total nitrogen (TN), total phosphorus (TP), and available nitrogen (AN) contents while enhancing the overall growth quality of cut lilies. Given that alterations in soil nutrient status are closely linked to dynamic shifts in the rhizosphere microbiome, we further examined the relationships between rhizosphere soil physicochemical properties and microbial taxa across different treatments. Mantel tests and Spearman correlation analyses (Figure 2) revealed that bacterial communities were significantly and positively correlated with most soil nutrient indicators, including pH, TN, and AN (correlation coefficient *r* ≥ 0.2, *p* < 0.05), whereas fungal communities showed significant negative correlations with available phosphorus (AP) and total potassium (TK). Notably, the correlations between bacterial communities and TP as well as soil organic matter (OM) were stronger than those between fungal communities and AK, suggesting that bacteria may be more closely associated with soil nutrient dynamics than fungi in this system. These rhizosphere-level mechanisms are consistent with the previously observed agronomic benefits of inoculant A, including increased plant height, elevated green leaf ratio, and reduced leaf yellowing and abscission index.

**Figure 2:**
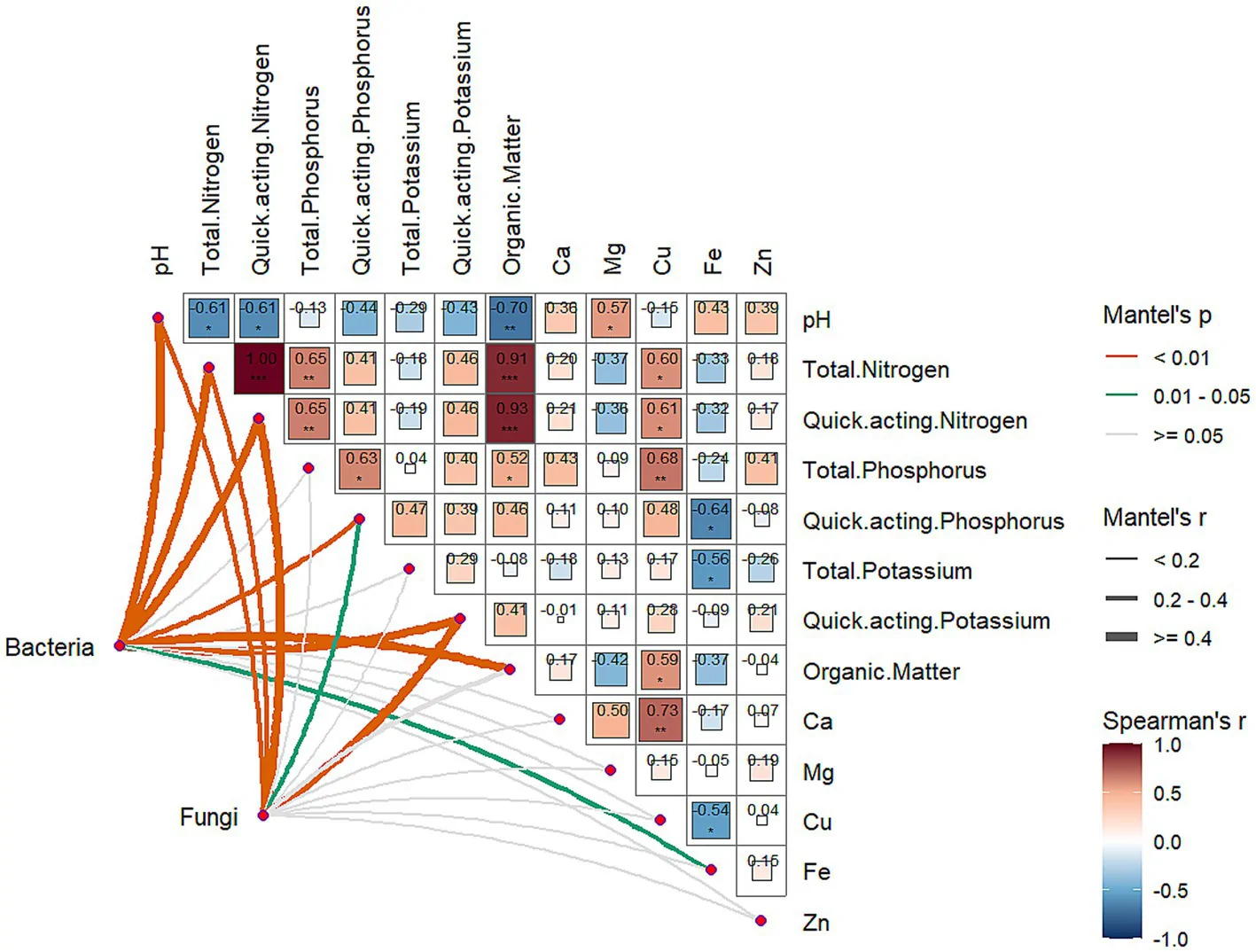
Combined Mantel test and Spearman correlation analysis of microbial community and soil physicochemical properties. Heatmap colors represent Spearman correlation coefficients (red: positive correlation; blue: negative correlation; darker color indicates stronger correlation). Left connecting lines are Mantel test results: orange line indicates *p* < 0.01, green line indicates 0.01 ≤ *p* < 0.05; line thickness represents Mantel correlation coefficient *r* (thicker line indicates larger *r*).

### 3.4 Effects of different inoculants on rhizosphere bacterial community structure

#### 3.4.1 Bacterial community diversity and structural differences

Based on alpha diversity analysis of rhizosphere soil bacterial community (Table 3), different inoculant treatments could significantly affect the richness and diversity of rhizosphere bacterial community (*p* < 0.05). In terms of species richness, the ACE and Chao1 indexes of Group C (8196.667 ± 429.826) were significantly superior to other treatments, indicating the highest species richness; Groups A and B had moderate richness; while CK control and BFP groups had the lowest richness. In terms of species diversity, Shannon index showed that Group C had the highest microbial community diversity (11.724 ± 0.170), followed by Group A (11.648 ± 0.178) and Group B (11.575 ± 0.282), and CK control (11.478 ± 0.160) and BFP groups (11.451 ± 0.059) had significantly lower diversity. Notably, Group C had the highest alpha diversity due to its composite microbial composition, but its leaf yellowing and abscission index was relatively high; while Group A had moderate diversity index, indicating that its disease control mechanism did not rely on maximizing species richness, but might achieve disease inhibition by regulating the composition of specific functional microbial groups.

**Table 3:** Alpha diversity indexes of rhizosphere bacterial community under different inoculant treatments.

Group	Sample size	ACE	Chao1	Shannon
A	6	7889.333 ± 950.631^b^	7889.333 ± 950.631^b^	11.648 ± 0.178^b^
B	6	7819.333 ± 619.680^b^	7819.333 ± 619.680^b^	11.575 ± 0.282^c^
C	6	8196.667 ± 429.826^a^	8196.667 ± 429.826^a^	11.724 ± 0.170^a^
CK	6	7600.333 ± 565.860^c^	7600.333 ± 565.860^c^	11.478 ± 0.160^d^
BFP	6	7461.667 ± 505.734^d^	7461.667 ± 505.734^d^	11.451 ± 0.059^d^

Values are mean ± standard deviation (*n* = 6 soil samples, each sample from one replicate plot). Different letters indicate significant differences among treatments (*p* < 0.05, Duncan’s test). ACE and Chao1 indexes estimate species richness; Shannon index reflects species diversity. During quality filtering, singleton ASVs were removed; therefore, the F1 values for all samples are 0, and both Chao1 and ACE richness estimators are reduced to observed ASV richness (S_obs).

Beta diversity analysis of rhizosphere soil bacterial community showed that principal coordinate analysis (PCoA) results (Figure 3A) revealed that two principal coordinates contributed 36.51% to the variation of soil microbial community, with PCO1 and PCO2 explaining 23.24 and 13.27% of the variation respectively, indicating that lily planting significantly changed soil bacterial community structure, and the addition of different microbial inoculants had a weak but detectable effect on community structure. Intergroup difference analysis by non-metric multidimensional scaling (NMDS), Tukey HSD test and analysis of similarities (ANOSIM) test (Figures 3B,C) further verified the above conclusion. The Bray–Curtis heatmap further illustrated distinct clustering patterns for each treatment (Figure 3D), showing significant differences in bacterial community structure among different treatment groups. Inoculant treatment groups A, B and C were clearly separated from CK and BFP groups, among which Group A had high sample aggregation, indicating stable bacterial community structure, while Group B partially overlapped with Groups A and C with weak regulatory specificity.

**Figure 3:**
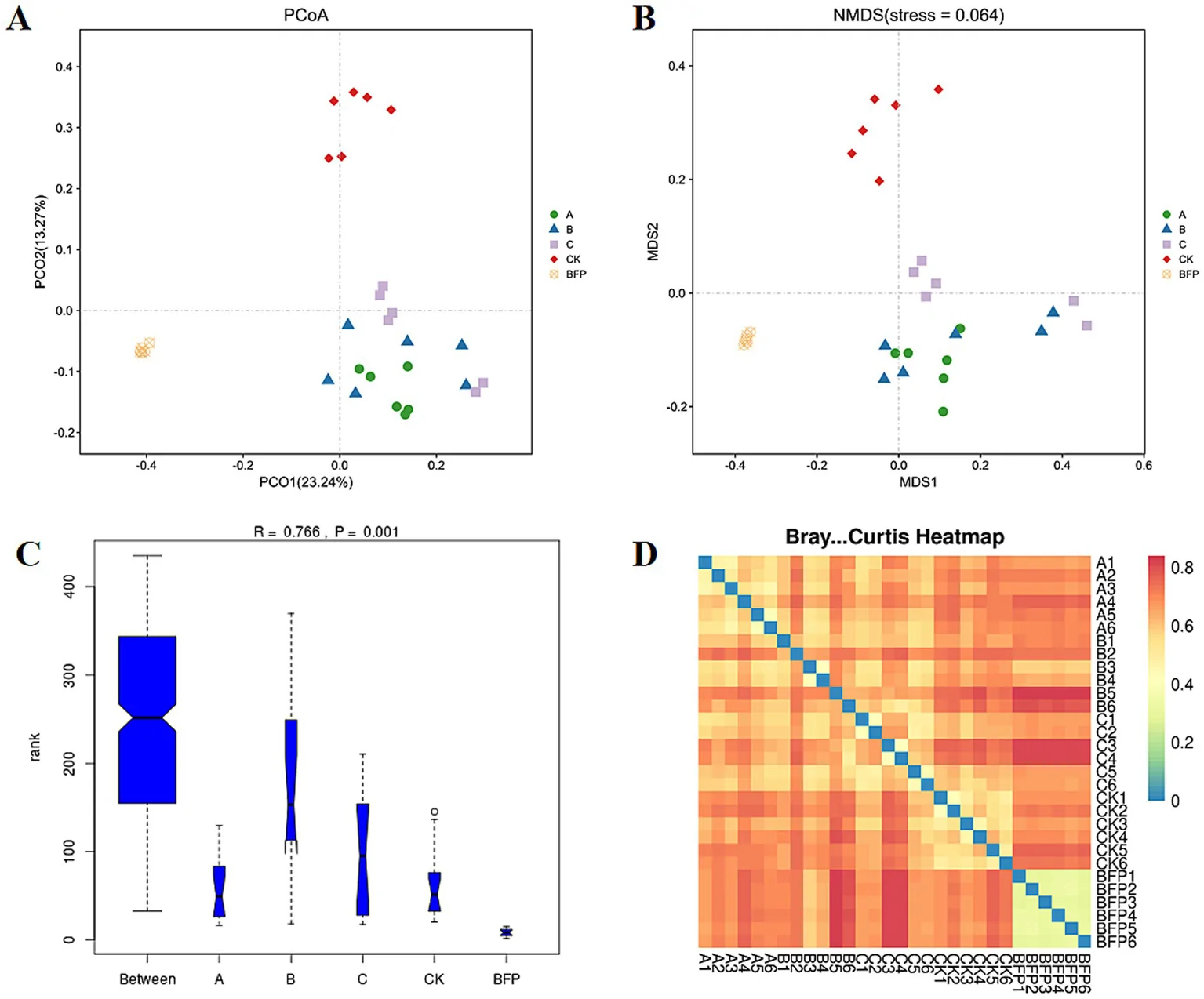
Beta diversity analysis of rhizosphere bacterial community of lilies under different inoculant treatments. **(A)** Principal coordinate analysis (PCoA) based on Bray-Curtis distance; **(B)** Non-metric multidimensional scaling (NMDS); **(C)** Anosim test (*R* = 0.766, *p* = 0.001); **(D)** Bray-Curtis distance heatmap illustrating the dissimilarity matrix among samples. Each point represents one soil sample (*n* = 6 per treatment). Different colors represent different treatments (see legend). NMDS stress = 0.064. Anosim based on 999 permutations.

#### 3.4.2 Bacterial community composition and screening of key microbial communities

Based on clustering with 97% sequence similarity, ASV analysis was performed using detection results from soils treated with different microbial agents. Venn diagrams and Upset analyses revealed distinct species compositions among rhizosphere bacterial communities across treatment groups, encompassing both shared and unique species (Figures 4A,B), with the presence of unique species serving as a key factor contributing to structural differences. A total of 1,014 shared ASVs were identified across all soils, indicating stable core bacterial communities under different microbial treatments. Additionally, significant numbers of unique bacterial OTUs were detected in each group: 4,712 unique in Group A, 5,049 in Group B, 5,100 in Group C, 5,066 in the CK control group, and 5,152 in the BFP group. These findings demonstrate that microbial agents significantly alter soil bacterial community composition by inducing the emergence of numerous unique bacterial taxa, with varying scales of enriched distinctive communities across treatments. The results vividly illustrate both similarities and differences in bacterial community structures among samples, providing robust evidence for understanding the effects of different treatments on soil microbial community architecture.

**Figure 4:**
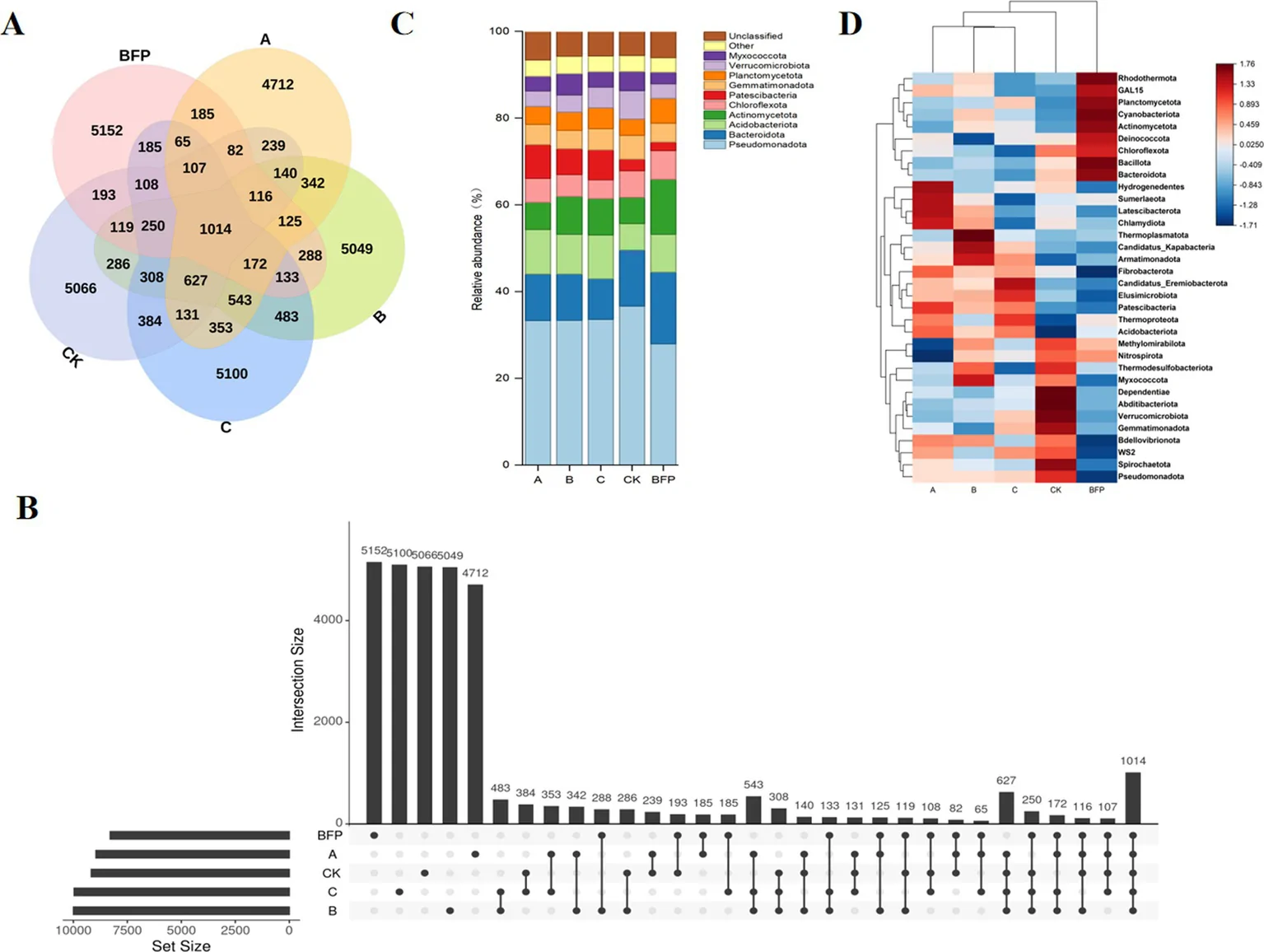
Rhizosphere bacterial community composition of lilies under different inoculant treatments. **(A)** Venn plot (showing shared and unique ASVs); **(B)** Upset plot (ASV intersection); **(C)** Phylum-level relative abundance (stacked bar chart); **(D)** Phylum-level relative abundance heatmap (darker color indicates higher relative abundance, red for high abundance, blue for low abundance). ASVs were clustered at 97% sequence similarity. Only phyla with average relative abundance >1% in all samples are shown.

Phylum-level community composition analysis (Figure 4C) showed that dominant phyla of lily rhizosphere bacteria included Pseudomonadota, Actinomycetota, Acidobacteriota, Bacteroidota, Chloroflexota, Planctomycetota, Gemmatimonadota, Patescibacteria, Myxococcota, Verrucomicrobiota, etc., accounting for more than 90% in total. Different inoculant treatments had differential regulatory effects on the relative abundance of dominant phyla: Treatment A significantly increased the relative abundance of Pseudomonadota and Actinomycetota; Treatment C had more prominent enrichment effect on Planctomycetota and Gemmatimonadota; Treatment B mainly regulated the relative abundance of Bacteroidota and Chloroflexota; while CK and BFP groups had higher relative abundance of Acidobacteriota and Patescibacteria. Phylum-level heatmap analysis further revealed the differential characteristics of bacterial community among different treatment groups (Figure 4D): Pseudomonadota and Actinomycetota were highly expressed (red) in Groups A and C; Acidobacteriota and Patescibacteria were highly expressed in CK and BFP groups; Chloroflexota and Planctomycetota were highly expressed in Group C, indicating that different inoculants could directionally regulate phylum-level microbiota structure and further change rhizosphere microecological environment. Genus-level community composition analysis (Supplementary Figure S2A) showed that dominant genera of lily rhizosphere bacteria included *Pontibacter*, *Gemmatimonas*, *Bryobacter*, *Pseudolabrys*, *Opitutus*, *Rhodanobacter*, *Chryseolinea*, *Dongia*, *Aggregatilinea*, *Sphingomonas*, etc. Different inoculant treatments had obvious differential enrichment effects on dominant genera. Genus-level heatmap analysis (Supplementary Figure S2B) further clearly showed the differential expression characteristics of microbiota in each group: Treatment A directionally enriched genera such as *Pseudomonas* and *Streptomyces*, both of which contain species with reported plant-beneficial functions. *Pseudomonas* has been reported to secrete antibiotics and activate soil phosphorus; *Streptomyces* has been reported to decompose soil OM and promote nitrogen cycling. The enrichment of these two putatively beneficial genera may contribute to the optimization of soil nutrients and improvement of lily stress resistance under Treatment A. Some potentially detrimental taxa showed higher abundance in CK and BFP groups, indicating that inoculant treatment could optimize rhizosphere bacterial community structure by enriching beneficial genera and inhibiting harmful genera.

### 3.5 Effects of different inoculants on rhizosphere fungal community structure

#### 3.5.1 Fungal community diversity and structural differences

Alpha diversity analysis of rhizosphere soil fungal community (Table 4) showed that different inoculant treatments significantly changed lily rhizosphere fungal community structure. In terms of species richness, ACE and Chao1 indexes showed that Group C had the highest fungal species richness, significantly higher than other treatments; CK and A groups were moderate with no significant difference; Group B had lower richness. In terms of species diversity, Shannon index showed a different trend: Group C had the highest diversity; Groups A and CK were moderate; Group B had the lowest diversity. Notably, Group A had moderate alpha diversity indexes of fungi, while Group C had the highest fungal richness but did not show the best effect on leaf yellowing and abscission index, indicating that the disease inhibition mechanism of Treatment A was not achieved by maximizing the species richness or diversity of rhizosphere fungal community, but might exert disease inhibition by regulating the abundance of specific beneficial fungal groups or the functional structure of fungal community.

**Table 4:** Alpha diversity indexes of rhizosphere fungal community under different inoculant treatments.

Group	Sample size	ACE	Chao1	Shannon
A	6	402.500 ± 38.647^b^	402.500 ± 38.647^b^	5.352 ± 0.768^b^
B	6	369.333 ± 79.847^c^	369.333 ± 79.847^c^	4.954 ± 1.002^c^
C	6	439.333 ± 95.447^a^	439.333 ± 95.447^a^	5.609 ± 0.734^a^
CK	6	415.167 ± 39.078^b^	415.167 ± 39.078^b^	5.296 ± 0.403^b^
BFP	6	304.333 ± 32.732^d^	304.333 ± 32.732^d^	5.545 ± 0.273^a^

Values are mean ± standard deviation (*n* = 6 soil samples, each sample from one replicate plot). Different letters indicate significant differences among treatments (*p* < 0.05, Duncan’s test). ACE and Chao1 indexes estimate species richness; Shannon index reflects species diversity. During quality filtering, singleton ASVs were removed; therefore, the F1 values for all samples are 0, and both Chao1 and ACE richness estimators are reduced to observed ASV richness (S_obs).

Beta diversity analysis of rhizosphere soil fungal community showed that PCoA results (Figure 5A) revealed that two principal coordinates contributed 31.85% to the variation of soil microbial community, with PCO1 and PCO2 explaining 22.30 and 8.55% of the variation respectively, indicating that lily planting significantly changed soil fungal community structure, and the addition of different microbial inoculants had a weak but detectable effect on community structure. Intergroup difference analysis by NMDS, Anosim test further verified the above conclusion (Figures 5B,C). The Bray-Curtis heat map reveals distinct clustering patterns among the different treatment groups (Figures 5D), showing significant differences in fungal community structure among different treatment groups. Inoculant treatment groups A and C were clearly separated from CK and BFP groups, among which Group A had the highest sample aggregation and the most stable community structure, while Group B had high sample dispersion and unstable regulatory effect.

**Figure 5:**
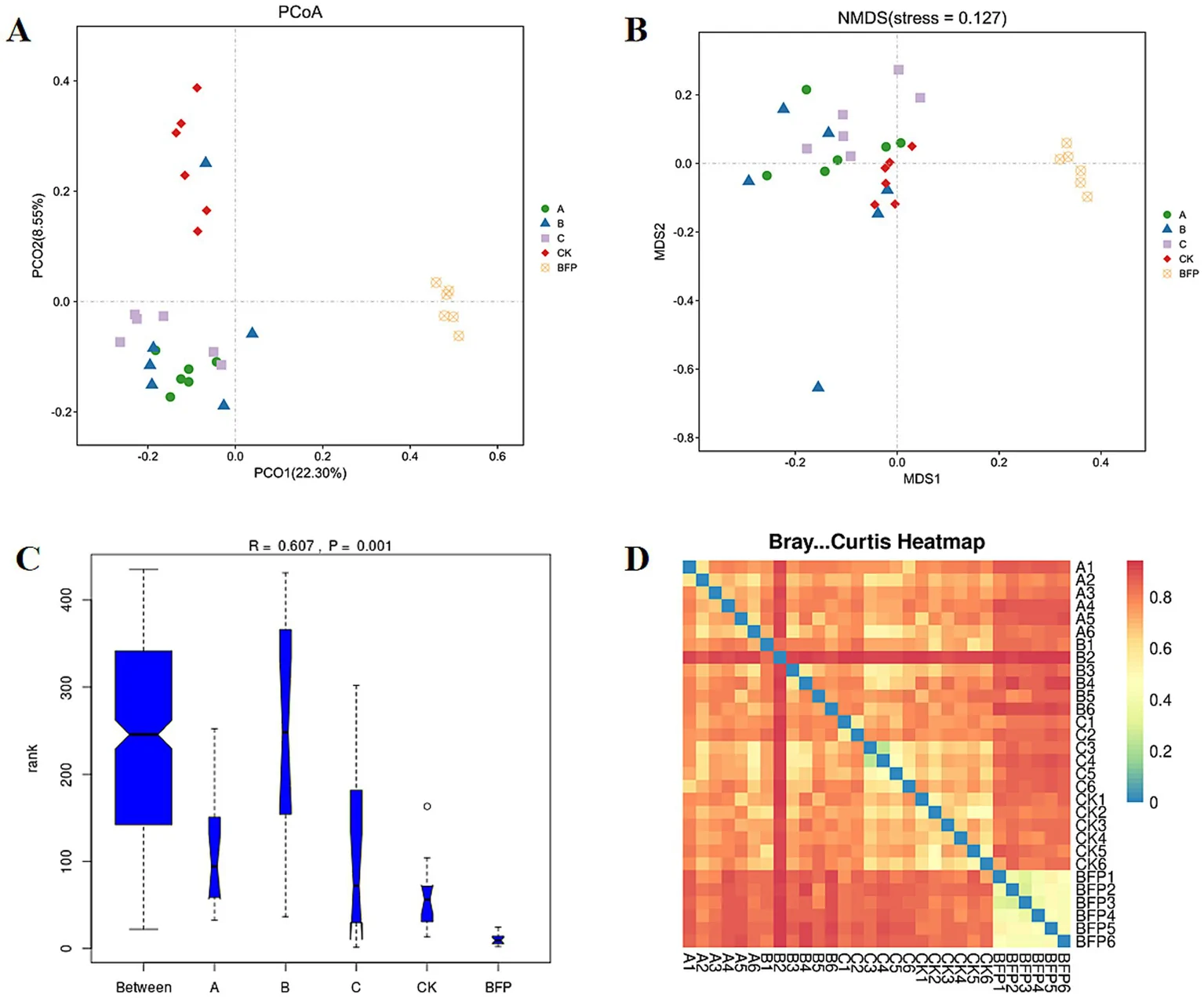
Beta diversity analysis of rhizosphere fungal community of lilies under different inoculant treatments. **(A)** Principal coordinate analysis (PCoA) based on Bray-Curtis distance; **(B)** Non-metric multidimensional scaling (NMDS); **(C)** Anosim test (*R* = 0.607, *p* = 0.001); **(D)** Bray-Curtis distance heatmap illustrating the dissimilarity matrix among samples. Each point represents one soil sample (*n* = 6 per treatment). Different colors represent different treatments (see legend). NMDS stress = 0.127. Anosim based on 999 permutations.

#### 3.5.2 Fungal community composition and screening of key microorganisms

Based on clustering with 97% sequence similarity, ASV analysis was performed using detection results from soils treated with different microbial agents. Venn diagrams and Upset analyses revealed differences in species composition among rhizosphere fungal communities across treatment groups, including both shared and unique species (Figures 6A,B), with the presence of unique species being a key factor contributing to structural variations. A total of 180 shared ASVs were identified across all soils, constituting the core shared taxa within this microbial community framework; 383 unique ASVs were detected in BFP treatment (soils without lily cultivation); 464 unique ASVs in agent A treatment; 434 in agent B treatment; and 489 in agent C treatment. Higher numbers of unique species were observed in agent A and C treatment groups, while greater shared species were present in agent B treatment and both CK and BFP groups, indicating that agents A and C significantly alter fungal community composition, whereas agent B exhibits weaker regulatory effects. The overall number of unique OTUs in fungal groups was lower than that in bacterial groups, suggesting higher stability of fungal communities compared to bacterial ones. Different microbial treatments still enriched substantial unique fungal taxa, demonstrating that microbial application substantially modifies soil fungal community composition, with varying enrichment scales observed across treatments.

**Figure 6:**
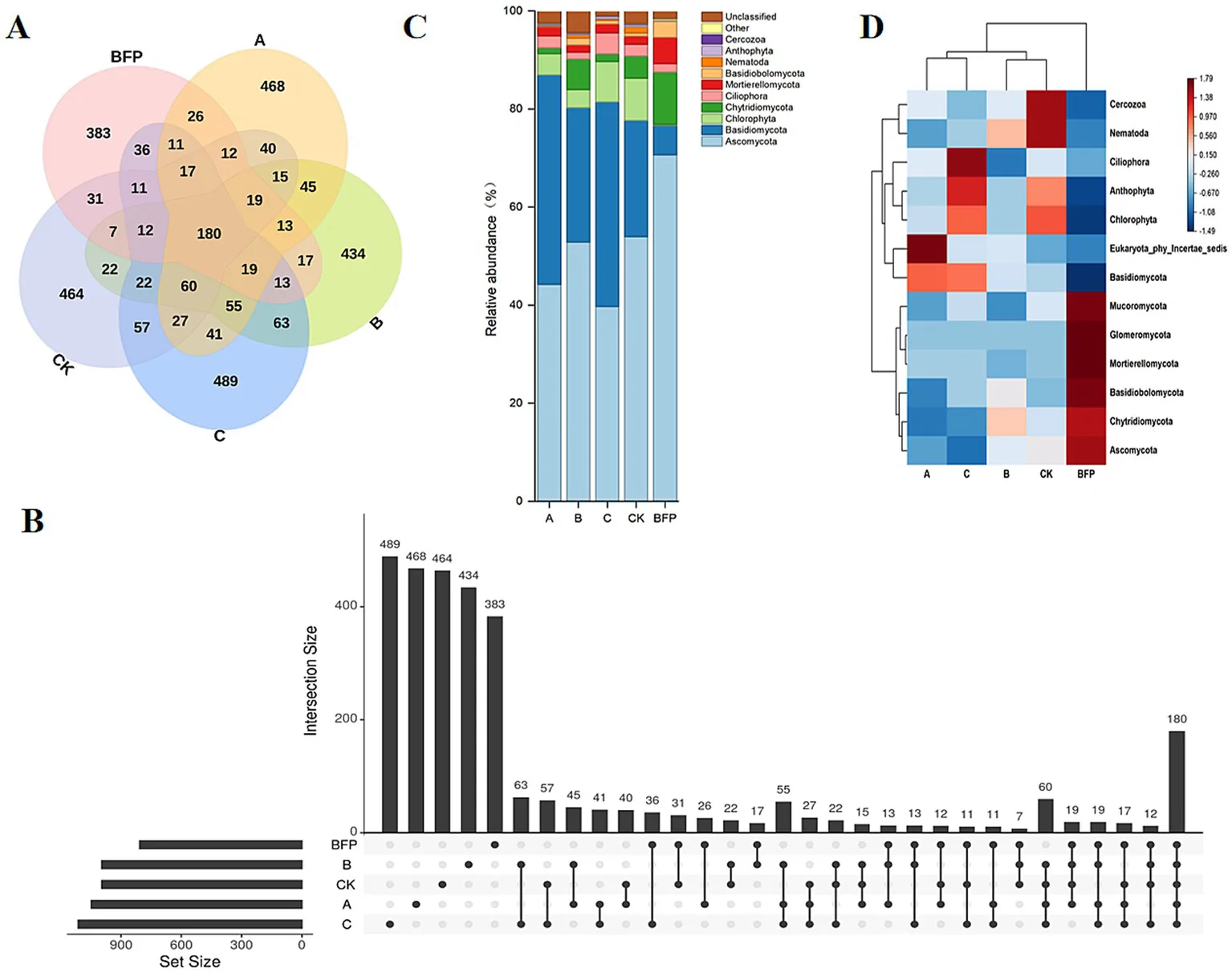
Rhizosphere fungal community composition of lilies under different inoculant treatments. **(A)** Venn plot (showing shared and unique ASVs); **(B)** Upset plot (ASV intersection); **(C)** Phylum-level relative abundance (stacked bar chart); **(D)** Phylum-level relative abundance heatmap (darker color indicates higher relative abundance, red for high abundance, blue for low abundance). ASVs were clustered at 97% sequence similarity. Only phyla with average relative abundance >1% in all samples are shown.

Phylum-level community composition analysis showed that dominant phyla of lily rhizosphere fungi included *Ascomycota*, *Basidiomycota*, *Mucoromycota*, etc., accounting for more than 80% in total (Figures 6C,D). Treatment A significantly increased the relative abundance of *Ascomycota* and *Mucoromycota* (*p* < 0.05) and inhibited the relative abundance of *Basidiomycota*; Treatment C mainly enriched *Mortierellomycota* and *Ascomycota*; Treatment B had no significant regulatory effect on dominant phyla, with little difference from CK group. Genus-level community composition analysis (Supplementary Figures 3A,B) showed that dominant genera of lily rhizosphere fungi included various functional fungi and pathogens. Treatment A significantly enriched putatively beneficial fungi such as *Trichoderma* and *Mucor*, and reduced the relative abundance of potentially pathogenic taxa such as *Fusarium* and *Rhizoctonia*; Treatment C mainly enriched beneficial fungi such as *Mortierella*; Treatment B had weak enrichment effect on beneficial fungi, and pathogen relative abundance had no significant difference from CK group.

Treatment A significantly increased the relative abundance of *Ascomycota* and *Mortierellomycota* by applying exogenous inoculant, optimized the functional structure of rhizosphere fungal community, significantly reduced leaf yellowing and abscission index and improved lily plant height and stem diameter. The relative increase of Mortierellomycota and Glomeromycota was consistent with the improvement of soil physicochemical indexes such as AN and OM.

### 3.6 Analysis of interactions in rhizosphere bacterial-fungal co-occurrence networks

#### 3.6.1 Response of rhizosphere soil bacterial-fungal co-occurrence networks to inoculants

To reveal the regulatory mechanism of different inoculants on rhizosphere microbial interactions, treatment-specific bacterial and fungal co-occurrence networks were independently constructed using the SparCC algorithm, which is specifically designed for compositional sequencing data and is well suited to low-sample, high-dimensional microbiome datasets. Given the modest replication per treatment (n = 6), these networks are interpreted as exploratory and topological differences are presented as descriptive trends.

Network analysis results showed significant differences in bacterial-fungal network structure under different inoculant treatments (Figures 7A,B). In bacterial network, Treatment A exhibited the highest modularity (*Q* = 0.4200), indicating clearer ecological functional differentiation of its community. Meanwhile, the proportion of positive correlations in Treatment A reached 88.79%, significantly higher than other treatment groups, while the proportion of negative correlations was only 16.14%, the lowest among all treatment groups. Network density and average degree analysis showed that the bacterial network density (0.0557) and average degree (4.96) of Group A were moderate, ensuring sufficient connection complexity while avoiding ecological instability caused by excessive connection. In Treatment A, Pseudomonadota nodes were not only dominant in quantity, but also formed extensive connections with Bacteroidota, Acidobacteriota, Actinomycetota, etc. Actinomycetota changed from marginal groups to key nodes, indicating the core role of *Trichoderma* and its metabolites in promoting fungal-bacterial and bacterial-bacterial synergy. These results indicated that Treatment A effectively promoted the synergistic interaction of beneficial bacteria and inhibited the spread of harmful groups. In fungal network, Treatment A also showed high modularity (*Q* = 0.3124) and proportion of positive correlations (72.35%), further confirming the optimization effect of Treatment A on fungal community structure.

**Figure 7:**
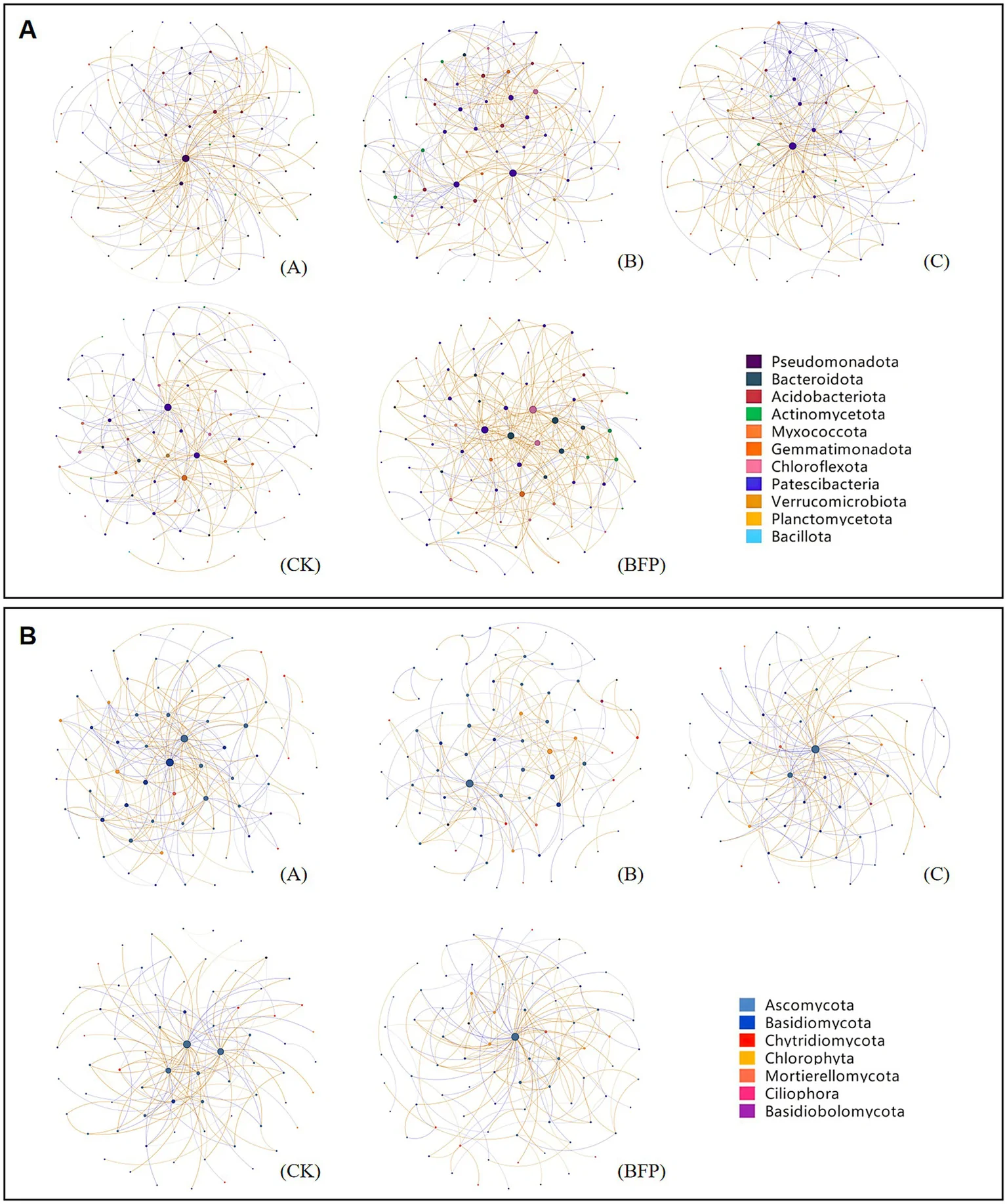
Treatment-specific rhizosphere soil bacterial and fungal co-occurrence networks under different inoculant treatments. **(A)** Bacterial co-occurrence networks; **(B)** Fungal co-occurrence networks. Each treatment group (*n* = 6) was analyzed independently. Nodes are colored by phylum-level classification; edges represent significant SparCC correlations (|*r*| > 0.6, *p* < 0.01, two-sided *p* values estimated by bootstrap permutation), with red indicating positive and blue indicating negative associations. Only nodes with at least two connections are shown.

#### 3.6.2 Combined screening of bacterial-fungal community indicator species

To study the directional regulatory effects of different microbial inoculants on lily rhizosphere bacterial and fungal communities and identify core indicator groups driving microecological functional differentiation, significantly enriched differential microbial groups in each group were systematically analyzed based on LEfSe results. For bacteria, Treatment A specifically enriched candidate phyla, Acidobacteriota and related groups, while Pseudomonadaceae, Dokdonella and other functional bacteria were significantly enriched, which were characteristic high-abundance indicator bacteria of Treatment A with the highest LDA values among all groups, and these groups stably clustered in red branches in evolutionary cladograms. Treatment B took Gemmatimonadota-related groups as core indicator bacteria; the core indicator bacteria of Treatment C were Acidobacteriota-related groups, only involved in basic soil nutrient cycling; CK and BFP groups were dominated by oligotrophic indigenous bacteria such as *Candidatus_Rokubacteria* and *Parvibacter*, reflecting the basic characteristics of soil microbial community without inoculant treatment. For fungi, Treatment A significantly enriched *Trichoderma* and *Mucor*, which were core indicator fungi containing species with reported biocontrol, growth-promotion and nutrient activation functions (Supplementary Figures S4A,B), with LDA values significantly higher than other groups, forming independent red branches in evolutionary cladograms, and were landmark fungi for regulating rhizosphere microecology in Treatment A. Treatment B took *Mortierella* as characteristic indicator fungus; the core indicator fungi of Treatment C were *Conocybe apala* and *Conocybe pilosa* under *Bolbitiaceae* in *Basidiomycota*, as well as saprophytic fungi such as *Nectriaceae* in *Ascomycota*, only involved in basic soil saprophytic metabolism; in CK and BFP (blue) groups, the abundance of potentially pathogenic taxa such as *Fusarium* and *Rhizoctonia* (genera known to include lily pathogens) increased significantly which were characteristic groups with high LDA values in this group (Supplementary Figures S4C,D). Combined bacterial-fungal indicator species analysis showed that Treatment A constructed a synergistic functional module of “growth-promotion, disease-resistance and nutrient activation” with candidate phyla, *Trichoderma* and *Mucor* as the core. This module was consistent with the improvement of soil TN, AN, AP and OM contents, as well as the increase of lily plant height, stem diameter, bulb fresh weight and the decrease of leaf yellowing and abscission index. The results indicated that Treatment A improved soil physicochemical properties by directionally enriching functionally synergistic bacterial-fungal indicator species, optimizing community structure and enriching characteristic functional groups, thereby promoting plant growth, providing theoretical basis for subsequent field application.

## 4 Discussions

The results of this study showed that Shentai *Trichoderma* combination (Inoculant A) could significantly promote the growth of lily plant height and stem diameter, and effectively improve cut flower quality. Thick stem is a key trait for breaking resistance and extending vase life of cut flowers during postharvest storage and transportation, directly related to market value (Mao et al., 2025; van Doorn et al., 2011; Zhou et al., 2025). In agricultural production, such quality improvement means an increase in the first-grade product rate and output value per unit area. Under greenhouse cultivation, lilies treated with Inoculant A showed better growth, and the first-grade yield rate of cut flowers is expected to increase significantly. Meanwhile, the observed reduction in leaf yellowing and abscission index and the enrichment of biocontrol taxa (e.g., *Trichoderma*, *Pseudomonas*) under Inoculant A suggest that this treatment has the potential to reduce pesticide reliance, although this remains to be directly validated in future trials that include reduced-pesticide input treatments (Zhou et al., 2025).

Under the condition of solar greenhouse continuous cropping soil, few studies have systematically compared the synergistic regulatory effects of three commercial microbial inoculants on the growth of Lilium ‘Siberia’, soil physicochemical properties, and rhizosphere bacterial-fungal community. This study addresses this gap by integrating analyses of inoculant formulation, rhizosphere microecological mechanisms, and commodity quality improvement in cut lily production. Existing microbial studies on flowers mostly focus on single strain or single index. Through multi-treatment and integrated multi-dimensional analysis combined analysis, this study confirmed that different inoculants have significantly differentiated action targets: Treatment A, with *Trichoderma* and its metabolites as the core, focuses on the synergistic improvement of growth-promotion, disease-resistance and nutrient activation; Treatments B and C focus on OM and AK enrichment and microbial community diversity improvement respectively, but fail to form functional links, so their comprehensive performance is inferior to Treatment A.

Functional analysis of key bacterial groups showed that the enrichment of *Pseudomonas* and *Streptomyces* was an important support for Inoculant A to optimize soil nutrients. Organic acids secreted by *Pseudomonas* can reduce soil pH and activate inert phosphorus in soil, increasing AP content (Zhao et al., 2024). *Streptomyces* can produce cellulase, protease, etc., accelerate soil OM decomposition, promote nitrogen cycling, and improve soil AN content (Liu et al., 2026a; Sun et al., 2025). The synergistic effect of these two genera is consistent with the significant improvement of soil OM, AN and AP contents by Inoculant A in this study (Wen et al., 2024). The high abundance of Acidobacteriota and Patescibacteria in CK group may be related to soil nutrient deficiency—most of these microbiota are oligotrophic microorganisms, and their enrichment often indicates the imbalance of rhizosphere nutrient cycling (Zheng et al., 2025), further confirming the regulatory pathway of Inoculant A to optimize soil nutrient cycling by enriching beneficial bacteria.

In fungal community, the enrichment of *Trichoderma* and *Mucor* was the key for Inoculant A to improve lily stress resistance. As a well-known rhizosphere growth-promoting fungus, *Trichoderma* hyphae can penetrate into OM and insoluble phosphate particles, and dissolve fixed phosphorus by secreting organic acids, phosphatase and other metabolites (Woo et al., 2023). Meanwhile, *Trichoderma* has good nitrogen fixation ability or can synergize with nitrogen-fixing microorganisms, thereby improving soil nitrogen level (Guzmán-Guzmán et al., 2025; Xin et al., 2023). *Trichoderma* has been reported to inhibit the colonization of potentially pathogenic taxa such as *Fusarium* and *Rhizoctonia* by competing for nutrient sites and producing chitinase to degrade fungal cell walls (Chen et al., 2025; Sun et al., 2024), which is consistent with the reduced leaf yellowing and abscission index observed under Inoculant A. *Mucor* can promote plant root development, improve root nutrient absorption efficiency, and synergistically decompose soil OM to further optimize soil fertility (Whalen et al., 2024), corresponding to the significant improvement of lily bulb fresh weight and flower bud number (Wang et al., 2025). Inoculant B contains multiple strains but fails to enrich core fungal groups; Inoculant C is mainly *Bacillus subtilis* with insufficient targeted regulation on fungal community, so neither can achieve synchronous improvement of stress resistance and growth potential.

The application of Inoculant A not only directly introduced beneficial *Trichoderma*, but also significantly changed the structure and interaction of indigenous rhizosphere microbial community. The bacterial and fungal networks constructed separately for the Inoculant A treatment had high node number, connection number and modularity. A more complex and stable microbial network means higher functional redundancy and ecological resilience (Wagg et al., 2019). Specifically, when the soil environment is stressed or invaded by pathogens, the complex network structure can maintain the stability of ecosystem functions through multiple alternative pathways. In bacterial community, Inoculant A treatment enriched Actinomycetota with growth-promotion and disease-resistance functions. Actinomycetes are known important producers of antibiotics, which can effectively inhibit a variety of soil-borne pathogens (Guo et al., 2016). Network analysis showed that Actinomycetota formed a positive correlation connection with Pseudomonadota (another important rhizosphere beneficial bacterium), and this strong correlation link may constitute a microecological barrier to inhibit pathogens. In the fungal community, Inoculant A treatment significantly reduced the relative abundance of *Fusarium*, a genus known to contain species that cause lily root rot and wilt (Chen et al., 2025; Sun et al., 2024), and weakened its connections in the co-occurrence network. The reduced abundance of this potentially pathogenic genus is consistent with the lower leaf yellowing and abscission index observed in this treatment. Therefore, Inoculant A optimized rhizosphere microecology through multiple pathways of “introducing beneficial microbes → activating indigenous beneficial microbiota → inhibiting pathogens,” creating a favorable environment for the healthy growth of lilies. Co-occurrence networks were constructed using SparCC, which is specifically designed for compositional microbiome data and has been shown to outperform conventional correlation methods in low-sample, high-dimensional settings. Nevertheless, as with any correlation-based network inference, the topological patterns reported here should be interpreted as exploratory given the modest replication (*n* = 6 per treatment), and future studies with larger sample sizes are encouraged to validate these findings.

From the perspective of industrial application, the results of this study can directly serve the green production of Lingyuan and northern lily main producing areas. Applying Shentai *Trichoderma* combination inoculant on the basis of conventional fertilization stably improved commodity traits such as cut flower plant height and stem diameter and reduced leaf yellowing and abscission (Zeng et al., 2025). Given that Inoculant A also enhanced soil nutrient availability (TN, AN, AP, OM) and enriched nutrient-cycling and biocontrol microbiota, it may offer a pathway toward reduced fertilizer and pesticide inputs in protected floriculture; however, dedicated reduced-input trials are needed to quantify the extent of any such reductions before this can be recommended as a standard practice (Yang et al., 2009; Mayak et al., 2004). Meanwhile, the putatively beneficial taxa such as *Pseudomonas*, *Streptomyces*, *Trichoderma* and *Mucor* identified in this study can serve as candidate strains for the construction of special synthetic microbial communities (SynCom) for lilies, providing targets for the research and development of precise and customized biological inoculants.

This study clarified the application effect of Inoculant A and preliminarily explained its microecological mechanism, but there are still deficiencies. Firstly, we did not measure soil enzyme activities related to nutrient cycling (e.g., urease, phosphatase) and disease resistance (e.g., chitinase), nor reveal changes in functional genes through metagenomics and other techniques, which makes the inference of microbial functions still at the correlation level. Future studies should combine metagenomics, metatranscriptomics and other techniques to directly verify the expression levels of related functional genes. Secondly, strain-level tracking was not performed; thus, the increased *Trichoderma* abundance detected by sequencing may reflect colonization by the inoculated *T. harzianum* strain, proliferation of indigenous *Trichoderma* taxa stimulated by the inoculant, or both. Strain-specific qPCR or marker gene tracking would be needed to distinguish these sources. In addition, this study was conducted as a single-season greenhouse trial under one soil type; field validation across different soil conditions and continuous multi-season monitoring are needed to assess the stability and broader applicability of Inoculant A. Furthermore, treatments with different application concentrations, periods, and methods were not included. The optimal field application scheme of Inoculant A still needs further optimization. Follow-up studies can carry out multi-factor orthogonal tests to clarify the key parameters of inoculant application, form standardized operating procedures that can be directly implemented, and promote the efficient transformation of scientific research achievements into large-scale production.

## 5 Conclusion

Integrating the above results, a synergistic regulation model of “inoculant—rhizosphere microbe—soil physicochemical property—lily growth” was constructed: Inoculant A (*Trichoderma* combination) was associated with the enrichment of key bacteria such as *Pseudomonas* and *Streptomyces*, as well as increased abundance of *Trichoderma* and *Mucor*, following the introduction of *Trichoderma harzianum* and its metabolites. These beneficial microbiota synergistically decomposed soil OM, activated AN and AP, adjusted soil pH to an appropriate range, and reduced the relative abundance of potentially pathogenic taxa such as *Fusarium*. The optimization of soil physicochemical properties further provided suitable conditions for the colonization of beneficial microbiota and lily growth, finally achieving significant improvement of lily growth indexes such as plant height, stem diameter and bulb fresh weight, as well as significant reduction of leaf yellowing and abscission index, forming a virtuous cycle.

## Data Availability

The data presented in the study are deposited in the NCBI Sequence Read Archive (SRA) repository, accession number SRP737115.
