# Supplementary material for: Responses of cut-lily quality and rhizosphere soil microecology to different microbial inoculants
Source: Front Microbiol. 2026 Oct 6;17:1962117. doi: 10.3389/fmicb.2026.1962117 (PMC13680473; doi:10.3389/fmicb.2026.1962117)
Supplement: Supplementary file 1 [file Supplementary_file_1.DOCX]

Supplementary Material

# 1 **Supplementary Figure 1.** Effects of different treatments on lily bulb fresh weight (A) and circumference (B)

**Supplementary Figure 2.** Relative abundance of lily rhizosphere bacterial communities at genus level: (A) stacked bars; (B) heatmap

**Supplementary Figure 3.** Relative abundance of lily rhizosphere fungal communities at genus level: (A) stacked bars; (B) heatmap

A

B

C

D

**Supplementary Figure 4.** Differential indicator species (bacteria and fungi) identified by LEfSe analysis among treatments (A) LDA score plot of bacteria;(B) LDA score plot of fungi;(C) Evolutionary cladogram of bacteria; (D) Evolutionary cladogram of fungi.

| Date | CK | A | B | C |
| --- | --- | --- | --- | --- |
| Day 1 | Planted beds 31–33; 630 bulbs; irrigated thoroughly after planting. | Planted beds 1–9; 1890 bulbs; bulbs soaked in inducer 1 min; “Cicisheng” 1 kg/bed broadcast to soil; irrigated thoroughly. | Planted beds 10–18; 1890 bulbs; “Mujianda” 3.3 kg/bed (total 30 kg) mixed into soil as basal fertilizer; bulbs soaked in “Junyangyuan” 100 g diluted solution 3 min; irrigated thoroughly. | Planted beds 19–30; 2520 bulbs; irrigated thoroughly; “Yingfeng” microbial agent 100 g/bed (total 1200 g) applied via drip irrigation. |
| Day 3 | Second irrigation, 5 min. | Second irrigation, 15 min. | Second irrigation, 15 min. | Second irrigation, 15 min. |
| Day 9 | West beds loosened. | West beds loosened. | West beds loosened. | West beds loosened. |
| Day 10 | — | — | — | Irrigated with microbial agent 1200 g; approx. 20 min; approx. 1 t water. |
| Day 17 | Irrigated with sugar alcohol calcium 0.5 kg; 8 min. | Irrigated with sugar alcohol calcium 0.165 kg/bed (1.5 kg for 9 beds); 20 min; approx. 1 t water. | Irrigated with sugar alcohol calcium 0.165 kg/bed (1.5 kg for 9 beds); 20 min; approx. 1 t water. | — |
| Day 19 | — | Amino acid foliar fertilizer, 3/4 bag per sprayer. | Amino acid foliar fertilizer, 3/4 bag per sprayer. | Amino acid foliar fertilizer, 3/4 bag per sprayer; microbial agent 1200 g and sugar alcohol calcium 2.0 kg applied with irrigation (same date as recorded). |
| Day 31 | — | Irrigated with “Tuwei No. 1” 0.5 bag and high-N bucket fertilizer 2.5 kg; approx. 20 min. | Irrigated with “Junyangyuan” 150 g and high-N bucket fertilizer 2.5 kg; approx. 20 min. | Irrigated with microbial agent 1800 g and high-N bucket fertilizer 2.5 kg. |
| Day 42 | Irrigated with iron 50 g and high-N bucket fertilizer 800 g. | — | — | Irrigated with microbial agent 1800 g, iron 350 g, and high-N bucket fertilizer 2.5 kg; 20 min. |
| Day 48 | — | Irrigated with iron 250 g, “Tuwei No. 1” 0.5 bag, and high-N bucket fertilizer 2.5 kg; approx. 20 min. | Irrigated with “Junyangyuan” 150 g, iron 250 g, and high-N bucket fertilizer 2.5 kg; approx. 20 min. | — |
| Day 57 | — | — | — | Irrigated with microbial agent 1800 g, iron 350 g, and high-N bucket fertilizer 2.5 kg; 20 min. |
| Day 65 | Irrigated with iron 50 g, high-K 1.5 kg, and high-N 1.0 kg. | Irrigated with iron 250 g, “Tuwei No. 1” 0.5 bag, and balanced fertilizer 1.5 kg; 20 min. | Irrigated with “Junyangyuan” 150 g, iron 250 g, balanced fertilizer 1.5 kg, and bucket K approx. 1.0 kg; 20 min. | — |
| Day 75 | — | — | — | Irrigated with microbial agent 1800 g, iron 500 g, and balanced fertilizer 2.0 kg; approx. 20 min. |
| Day 86 | Irrigated with high-K 0.75 kg and bucket K 1.0 kg. | — | — | — |
| Day 87 | — | Irrigated with “Cicisheng” 0.5 bag (500 g), balanced fertilizer 1.5 kg, and bucket K approx. 1.5 kg; 20 min. | Irrigated with “Junyangyuan” 150 g, balanced fertilizer 1.5 kg, and bucket K approx. 1.5 kg; 20 min. | — |
| Day 98 | Irrigated with balanced fertilizer 0.75 kg. | — | — | — |
| Day 102 | — | Irrigated with “Cicisheng” 0.5 bag, high-K 1.0 kg, and bucket K 1.5 kg; 20 min. | Irrigated with “Junyangyuan” 150 g, high-K 1.5 kg, and bucket K 1.5 kg; 20 min. | Irrigated with microbial agent 1800 g, high-K 2.0 kg, and bucket K approx. 2.0 kg; approx. 25 min. |
| Day 113 | Irrigated with high-K 0.75 kg. | — | — | — |
| Day 114 | — | Irrigated with high-K 2.0 kg and bucket K 1.5 kg; 20 min. | Irrigated with high-K 2.0 kg and bucket K 1.5 kg; 20 min. | Irrigated with microbial agent 1800 g and high-K 2.5 kg; approx. 20 min. |
| Day 122 | — | Irrigated with high-K 2.0 kg and bucket K 1.5 kg; 20 min. | Irrigated with high-K 2.0 kg and bucket K 1.5 kg; 20 min. | Irrigated with microbial agent 1800 g and high-K 2.0 kg; approx. 20 min. |

**Supplementary Table 1.** Field management records for CK and treatments A, B, and C.

**Note:** 1 jin = 500 g. “—” indicates no operation recorded on that date. All fertilizers and microbial agents were applied with irrigation unless otherwise stated. Pesticide management was not recorded in the original field logs. Where dilution ratio, exact irrigation volume, or product composition was not specified in the original records, this information should be verified from product labels and irrigation records before final submission.

**Abbreviations:** CK, control; A, treatment A; B, treatment B; C, treatment C; high-N, high-nitrogen fertilizer; high-K, high-potassium fertilizer; bucket K, bucket potassium fertilizer; approx., approximately; min, minute(s); t, tonne(s); g, gram(s); kg, kilogram(s).
